# Enhancing employees’ workplace well-being through workplace digitization: exploring the mediating roles of job demands and digital anxiety

**DOI:** 10.3389/fpsyg.2025.1642489

**Published:** 2025-07-25

**Authors:** Zhiyong Han, Guoqing Song, Yanlong Zhang, Lei Yan

**Affiliations:** ^1^School of Business Administration, Anhui University of Finance and Economics, Bengbu, China; ^2^School of Government, Central University of Finance and Economics, Beijing, China

**Keywords:** workplace digitization, workplace well-being, job demands, digital anxiety, the job demands-resources model

## Abstract

**Introduction:**

With the arrival of the digital era, workplaces are undergoing radical changes, and digitalization not only enhances work efficiency but also significantly influences employees’ workplace well-being. In this context, it is crucial for enterprises to actively promote digital transformation to gain a competitive edge. A significant challenge lies in liberating employees from monotonous tasks, thereby facilitating their engagement in more creative and analytical endeavors, which in turn enhances their workplace well-being. Consequently, investigating the mechanisms for improving workplace well-being during this transition has emerged as a critical area of inquiry in both scholarly and practical realms.

**Methods:**

Based on the Job Demands-Resources (JD-R) model, job demands and digital anxiety are introduced as mediating variables, and a dual-mediation model is constructed to explore the specific mechanism through which workplace digitization affects workplace well-being. A three-stage follow-up survey was conducted, resulting in 382 valid samples for rigorous statistical analysis.

**Results:**

The results show that workplace digitization positively impacts workplace well-being. Job demands and digital anxiety mediate the association between workplace digitization and workplace well-being. Additionally, workplace digitization reduces job demands and digital anxiety, which in turn enhances employees’ workplace well-being. These findings provide theoretical guidance on how management practices can enhance employees’ workplace well-being.

**Discussion:**

Based on the JD-R model, this study expands the research scope of workplace digitization outcome variables and enriches the antecedents of workplace well-being. By applying the JD-R model, this research positions workplace digitization as a macro-level organizational intervention that influences workplace well-being through both loss and gain pathways, thereby contributing valuable insights into its antecedents.

## 1 Introduction

During the ongoing wave of global digital advancement, digitization, networking, and intelligent systems have significantly deepened. The digital transformation of enterprises is becoming increasingly important, and enterprises must actively promote digital transformation if they wish to seize the first opportunity and gain an advantage in the fierce market competition (Wang and Zhang, 2024), and excellent talent capital is especially important in this process. During the implementation of digital transformation in enterprises, how to free enterprise employees from repetitive daily work, participate in more work that requires creativity, analysis and decision-making, and enhance employee workplace well-being has become an urgent issue for discussion in both academic and practical circles.

Zheng et al. (2015) define workplace well-being as employees’ overall positive evaluation of their work experience. Specifically, workplace well-being encompasses two core elements: job satisfaction and positive work-related emotions (Zheng et al., 2015). This dual construct emphasizes the integration of cognitive evaluation and affective experience, which is not only essential for organizational performance, but also a key indicator of organizational health (Renee Baptiste, 2008). The Global well-being Report 2023 states that with the passage of time, well-being has become a key indicator for countries and companies to detect. As work plays a central role in individuals’ lives, the organizational environment significantly influences employee well-being (Bakker and Demerouti, 2007). Specifically, unreasonable job demands and inappropriate leadership styles not only increase the workload of employees, but also reduce their job satisfaction, create burnout, and greatly reduce their workplace well-being (Peng et al., 2023). Therefore, investigating mechanisms for enhancing employee workplace well-being has profound theoretical and practical implications.

Regarding the concept of workplace digitization, Chan et al. (2021) draw on the UTAUT model constructed by Venkatesh and Davis (2003), which defines it as a process that involves changes in systems, processes, and roles within an organization, and is defined through the measurement of employees’ attitudes and sentiments towards digitization in the workplace (Ouyang et al., 2023). Consequently, achieving digital transformation within organizations greatly depends on how employees perceive and adopt digital technologies (Meske and Junglas, 2021). Recently, considerable academic discussion has focused on the mechanisms through which individual employee characteristics [e.g., employee proactive personality (Zhang et al., 2023)], job characteristics (e.g., job stress), and leadership factors [e.g., inclusive leadership style (Fang et al., 2022)] affect employees’ workplace well-being. Research investigating organizational digitization’s role in employees’ workplace well-being remains limited, warranting further investigation.

This study utilizes the JD-R model to explore the impact of workplace digitalization on workplace well-being. The JD-R model provides a systematic framework to analyze the contribution of job demands and resources to employee outcomes, serving as a comprehensive lens for examining the dual pathways through which digitalization influences workplace well-being. The model posits that job characteristics encompass two fundamental dimensions: job demands (aspects that require sustained effort and are associated with costs) and job resources (aspects that help achieve work goals, reduce demands, or stimulate personal growth) (Demerouti et al., 2001). This framework enables us to understand how digitalization simultaneously serves as a job resource while potentially altering traditional job demands, thereby creating multiple pathways to influence workplace well-being. The model’s dual-pathway mechanism provides valuable insights into how employees respond to the opportunities and challenges that digitalization presents in modern work environments.

Based on this framework, we argue that workplace digitalization affects workplace well-being through two key pathways aligned with the JD-R model’s dual-process mechanism. Following the job demands pathway, digitalization can reduce traditional job demands by automating repetitive tasks and streamlining work processes, thereby decreasing work-related strain and enabling employees to engage in more creative and meaningful work (Jia et al., 2024). Simultaneously, following the job resources pathway, digitalization functions as a workplace resource that can either strengthen employee competencies and reduce digital anxiety when well-implemented, or conversely, create digital anxiety when poorly aligned with employee needs and capabilities (Palumbo, 2022). Accordingly, this study examines two critical mediating mechanisms: job demands and digital anxiety, which correspond to the dual pathways of the JD-R model. By examining these two mediating pathways—job demands and digital anxiety—this study offers valuable insights into the ways in which digitalization influences workplace well-being through the complementary processes of demand reduction and resource optimization, offering both theoretical insights and practical guidance for successful digital transformation implementation.

## 2 Theoretical model and development of hypotheses

### 2.1 Theoretical models

**Figure 1:**
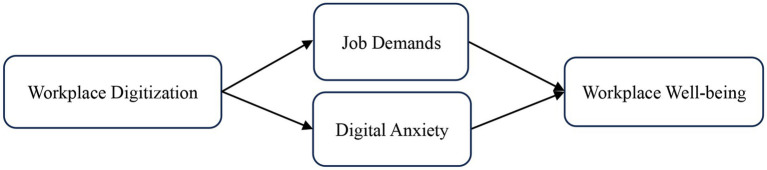
Theoretical model.

This study draws upon the JD-R model to explore the effects of workplace digitalization on employees’ workplace well-being. Demerouti et al. (2001) proposed the JD-R model, which posits that job characteristics can be classified into two primary categories: job demands and job resources. Job demands refer to the physical, psychological, social, and organizational aspects of work that require sustained effort, such as workload and time pressure. Job resources are aspects of work that can reduce the physical and psychological costs associated with job demands while stimulating personal growth and development. The job demands pathway (also known as the depletion pathway) suggests that excessive job demands deplete employees’ energy and resilience, leading to strain and ultimately resulting in negative outcomes such as burnout and reduced well-being (Bakker et al., 2004; Hakanen et al., 2006; Xanthopoulou et al., 2007). Conversely, the job resources pathway (also known as the gain pathway) indicates that adequate job resources enhance employee motivation, engagement, and positive work outcomes by helping individuals cope with demands and achieve their goals. These dual pathways have been extensively validated in various organizational contexts (Zhang et al., 2024; Hu et al., 2023; Bakker et al., 2023). A digital workplace alters employees’ original work styles and behaviors, which subsequently reduces job demands and increases job resources. This frees employees from daily repetitive tasks and activates the gain pathway and mitigates the depletion pathway, thereby enhancing employees’ well-being. According to Halbesleben et al. (2014), resources are things that individuals perceive as contributing to the achievement of their goals, depending on their subjective perceptions and assessments. It is emphasized that the significance of particular resources depends on how well they match relative to the individual’s current needs or goals. In the context of workplace digitalization, digital technologies represent valuable job resources. When digitalization aligns well with employees’ capabilities and work needs, employees perceive it as a beneficial resource that facilitates task completion and goal achievement, thereby reducing digital anxiety. This, consequently, will boost workplace satisfaction. Therefore, this study takes job demands and digital anxiety as mediating variables of workplace digitization affecting workplace well-being, and constructs a dual-mediation theoretical model. As depicted in Figure 1.

### 2.2 Workplace digitization and workplace well-being

Workplace digitization have led to significant changes in the way employees work, enabling them to access information and utilize social networks more efficiently, thereby increasing productivity, reducing work costs, and creating more value (Seeber and Erhardt, 2023; Hooi and Chan, 2023). By reviewing related literature, most studies on workplace digitalization focus on work design and organizational processes (e.g., Schwarzmüller et al., 2018), and few studies focus on the individual behavior of employees within the organization. Therefore, it is essential to examine how workplace digitization affects employees’ workplace well-being, considering the perspective of the employees themselves.

Workplace digitization is not only occurring but is also expanding exponentially (Ferraris et al., 2019; Bresciani et al., 2021). Specifically, workplace digitization refers to the systematic transformation of organizational systems, processes and roles achieved through technologies such as artificial intelligence, big data, and cloud computing. This change is not only transforming traditional work styles, employment relationships, and work practices, but it is also shaping the future of the workplace (Kumar et al., 2023; Ciasullo and Lim, 2022; Malhotra, 2021). Zheng et al. (2015) built upon Page and Vella-Brodrick's (2009) theoretical model to identify a three-dimensional structure of employee well-being: Life Well-being, Workplace Well-being, and Psychological Well-being. Zheng et al. (2015) define Workplace Well-being as an important dimension of employee well-being, incorporating both job satisfaction and emotional responses related to work. It is worth noting that digital transformation has a two-sided impact: On the one hand, the innovative capacity of the digitized workplace has been shown to enhance employee performance and improve work-life balance (Chatterjee et al., 2023); on the other hand, it may raise issues such as employee anxiety, privacy, compensation and benefits, well-being and job ambiguity (Acemoglu and Restrepo, 2020; Brougham and Haar, 2020; Makridis and Han, 2021; Santana and Cobo, 2020; Yu et al., 2022; Spencer, 2018). Consequently, it’s essential to direct attention to the factors influencing employee motivation, with a view to investigating the impact of workplace digitization on workplace well-being.

Determinants of workplace well-being fall into two groups: individual-level and organizational-level factors. Zou et al. (2015) argued that workplace well-being is not solely contingent on individual personality traits, but is also influenced by environmental factors, including work tasks and organizational context. Similarly, Fang et al. (2022) also asserted that the organizational environment is a crucial determinant of employees’ well-being in the workplace. In the context of digitization, Soga et al. (2022) found that digitized workplaces provide opportunities for learning and skill development, contributing to personal growth and development. These findings support the JD-R model, which indicates that increased job demands can impair employee health and well-being, whereas increased job resources provide motivational potential and promote work engagement (Bakker and Demerouti, 2007). When digitized workplaces reduce job demands by streamlining work processes and simultaneously enhance the organizational environment by providing learning opportunities and additional resources, they create favorable conditions for improving employee workplace well-being (Scholze and Hecker, 2024). Based on this theoretical framework, digital workplace transformation fundamentally alters work environments and methods, enabling employees to complete tasks more efficiently while simultaneously enhancing their emotional and workplace well-being. Accordingly, this study posits the following hypotheses:

*H1:* Workplace digitization positively impacts workplace well-being.

### 2.3 The mediating role of job demands

Digitization has not only altered the structure of workplaces and the nature of work itself but also transformed how employees engage in communication and learning within the workplace (Vallo Hult and Byström, 2022). In the near future, the digitization of work processes and practices may be reshaped through digital reforms within organizations, thus transforming the labor market for society as a whole (Lilja, 2020). For example, the use of technologies like mobile devices and information processing tools in the workplace frees employees from routine and repetitive tasks, thus changing the original design and structure of their work (Chan et al., 2021). By introducing digital channels and digital tools, the way employees work will change significantly. Employees can achieve a high level of connectivity at work, maintain flexibility and have easier access to information and make better use of social networks (Seeber and Erhardt, 2023). This will help reduce costs, save time, increase productivity and create more value (Hooi and Chan, 2023; Wisskirchen et al., 2017). Digital innovations and technological advances in the healthcare workplace enhance employee productivity, job satisfaction, and at the same time can reduce repetitive work tasks and reduce workplace hazards (Li et al., 2023). While digitization may temporarily increase skill learning requirements, it serves as a long-term job resource that reduces job demands and promotes employee development through workflow optimization and automation (Nadeem et al., 2024). Consequently, we propose the following hypotheses:

*H2a:* Workplace digitization has a negative impact on job demands.

The JD-R model posits that any given job encompasses two distinct dimensions: job demands and job resources. According to Demerouti et al. (2001), job demands encompass the physical, psychological, and social aspects of a job that require sustained effort, leading to associated physiological or psychological costs. Simply put, job demands encompass workplace stressors that deplete employee energy levels. The aforementioned factors encompass, but are not limited to, work overload, role conflict, time pressure, and job insecurity (Li and Xu, 2019). Previous research has consistently found negative effects of job demands. For instance, research demonstrates their association with negative health manifestations, notably burnout (Hakanen et al., 2006), where these demands are often cited as the primary contributors to burnout, resulting in diminished health and negative organizational consequences. High job demands can cause employees to develop emotional exhaustion, which reduces employee performance (Bakker et al., 2014). High workload demands frequently culminate in employees feeling overwhelmed and emotionally detached from their professional responsibilities (Bakker and Demerouti, 2017). When employees experience chronic fatigue and develop cynicism towards their jobs, they may subsequently report significant mental health issues (Toker and Biron, 2012). However, to meet job demands, employees must consistently engage in sustained effort. This process, however, depletes employees’ physical resources (e.g., physical strength), cognitive resources (e.g., attention), and mental energy (e.g., perseverance) (Bakker and de Vries, 2021). Once these corporeal and psychic resources are exhausted, job demands can shift from being mere requirements to becoming potential stressors or threats (Demerouti et al., 2001; Schaufeli and Bakker, 2004; Nauman et al., 2019), which reduces the employee’s work experience and ultimately leads to a decrease in employee workplace well-being. According to the preceding analysis, the study posits the following hypothesis:

*H2b:* Job demands negatively influence employee workplace well-being.

Combining hypotheses H2a and H2b, increased workplace digitization amplifies digital change’s influence on employee job demands, while simultaneously reducing job demands as employees adapt their work styles. When facing low job demands, employees do not need to consume more job resources, which leads to positive emotional feelings and enhances employees’ workplace well-being. Therefore, the study hypothesizes the following:

*H2:* Workplace digitization influences workplace well-being through the mediating role of job demands.

### 2.4 The mediating role of digital anxiety

Digital anxiety is a psychological phenomenon marked by adverse emotional states that result from the merging of digital technology with contemporary society. As the digital era progresses, these emotional states become increasingly persistent (Chen and Liu, 2024). Digital workplace anxiety is a psychological condition resulting from the gap between the rapid advancement of digital technology and individuals’ capacity to adapt effectively to their work environment. In the context of digital technological change, it is evident that employees will present a diverse range of psychological states. As organizations undergo digital transformation, employees may experience a high degree of uncertainty about future job demands, which may in turn trigger digital anxiety (Firk et al., 2023). They conceptualize digital anxiety as a cluster of adverse emotions such as apprehension, heightened arousal, and sometimes fear, which employees may experience digital anxiety when confronted with the widespread adoption and proliferation of digital technologies. The JD-R model emphasizes that job resources serve essential functions in facilitating work objectives and fostering individual advancement (Bakker and Demerouti, 2017). The increased resources brought by workplace digitization facilitate employees’ skill enhancement (Liu et al., 2024). Employees perceive these as beneficial resources that promote task completion and goal achievement, thereby reducing digital anxiety and generating more positive work behaviors (Chen et al., 2024). An analysis of 1,038 finance employees in a multinational business group indicates that higher levels of digital anxiety correspond to lower work engagement. Furthermore, the stronger the digital work atmosphere, the less digital anxiety employees feel within the organization (Firk et al., 2023). Accordingly, the study hypothesizes the following:

*H3a:* Workplace digitization negatively impacts digital anxiety.

In the midst of digital change in the workplace, employees may experience anxiety and restlessness, which may stem from unfamiliarity with new technologies and tools, uncertainty about future job requirements, and concerns about their own abilities and adaptability. With the deep integration of digital technology and business, employees face pressure to transform, and problems such as not daring to transform, not willing to transform, and not being good at transforming arise (Dang and Li, 2023). Furthermore, employees in the organization face a rapid surge in information, potentially leading to concerns like health anxiety (Muse et al., 2012). The JD-R model suggests that once job demands surpass available resources, employees experience strain and reduced well-being (Bakker and Demerouti, 2017). Digital transformation introduces new job demands that require continuous psychological and cognitive efforts to adapt to technological changes. When employees lack sufficient digital resources or support to meet these demands, digital anxiety emerges as a psychological strain response. Research shows that when employees hold negative attitudes toward technologies such as artificial intelligence, the resulting anxiety reduces their workplace well-being (Konuk et al., 2023). Similarly, technological stress and anxiety brought about by digitization have negative effects on employee workplace well-being (Marsh et al., 2024). Digital anxiety in the workplace may lead to stress, anxiety, and self-doubt among employees, which in turn affects their performance and mental health, thereby reducing their workplace well-being. Therefore, the study hypothesizes the following:

*H3b:* Digital anxiety negatively affects workplace well-being

Combining Hypotheses H3a and H3b, the more digitized the workplace is, the greater the impact of digital change on employees’ digital anxiety. Digital change in the workplace leads to a reduction in digital anxiety as employees become more confident in adapting to the digital work environment. When employees are less digitally anxious, they are more confident and capable of facing the challenges posed by digital technology, as well as better integrated and developed in the digital environment, enhancing their workplace well-being. In conjunction with JD-R model, a digital workplace increases the digital work atmosphere, which reduces employees’ digital anxiety and triggers the gain path, thus increasing employees’ workplace well-being. Therefore, the study hypothesizes the following:

*H3:* Digital anxiety acts as a mediator between workplace digitization and workplace well-being.

## 3 Method

### 3.1 Procedure and participants

A multi-temporal approach was used to collect data in this study from December 2023 to March 2024. The study employed the online survey platform Credamo to distribute questionnaires. This platform stands as one of China’s largest professional online survey platforms, having conducted more than 340,000 research projects with data quality that aligns with the criteria of international academic journals. To mitigate potential common method bias from affecting the study results, data collection occurred in three distinct stages. To mitigate the potential impact of consistency motivation and to prevent the subjects from becoming distracted, the questionnaire utilized a reverse scoring approach. During the first phase of data collection, participants evaluated workplace digitization and provided demographic variables and other personal information, including gender, age, education level, and department. Of the 600 questionnaires distributed, 512 responses were deemed valid, yielding a 85.3% response rate. In the second phase of the investigation, participants completed a questionnaire designed to evaluate job demands and digital anxiety. 426 valid questionnaires were obtained in total. In the third stage, subjects were invited to complete an evaluation of workplace well-being. Following the matching of the questionnaires from the three stages and the elimination of invalid samples, 382 valid samples were obtained, yielding a questionnaire response rate of 63.7%. The decision to employ multiple time points for data collection in this study was informed by the recognition that the impact of digital transformation on employees is a gradual process, and that it is challenging to accurately capture this dynamic change in a single point in time measurement. 382 valid samples were collected from the provinces of Anhui, Jiangsu, Shanghai, Zhejiang, and Shandong. These regions were primarily engaged in manufacturing (108, representing 28.3% of the total), information technology (82, accounting for 21.50%), high-tech and new technology (26, or 6.8%), finance, and financial services (6.8%). Additionally, the financial industry accounted for 20 samples (5.2%), among other sectors. These industries were chosen because they are at different stages of digital transformation, and this difference helps us to understand the impact of digitization on employees’ work experience more fully (Lu and Wang, 2021). The sample included 152 males (39.8%) and 230 females (60.2%). The largest proportion of respondents, comprising 203 individuals (53.1%), were aged between 26 and 35. The level of education was relatively high, with the majority having completed an undergraduate degree (268, 70.2%). Furthermore, the research samples are distributed widely in terms of region, balanced in terms of gender, and have a reasonable structure in terms of age and educational attainment, which ensures that the data samples are representative.

### 3.2 Measures

This research applied well-established scales developed by international scholars. The selected scales of workplace digitization, job demands, digital anxiety, and workplace well-being have been extensively employed in domestic research or have consistently demonstrated high reliability and validity. For each scale, the standard “translation-back-translation” procedure was employed, with reference to the Chinese translations of the relevant literature, and adjustments were made as needed to suit the local context. All the scales were scored using a 5-point Likert system, with subjects rating the descriptions of the items from 1 (representing “not at all conforming (disagreeing)”) to 5 (representing “fully conforming (agreeing)”).

#### 3.2.1 Workplace digitization

This study utilized the workplace digitization scale from Ouyang et al. (2023). This scale was revised by Ouyang et al. based on the workplace digitization scale developed by Chan et al. (2021). Chan et al. (2021) initially developed a 16-item workplace digitization scale based on Venkatesh and Davis’s (2003) Unified Theory of Acceptance and Use of Technology (UTAUT) model, reinterpreting the core dimensions of UTAUT (performance expectancy, effort expectancy, social influence, and facilitating conditions) as dimensions for measuring workplace digitization levels. Building on Chan et al.’s (2021) scale, Ouyang et al. (2023) deleted one item and revised and validated the scale for the Chinese context, ultimately creating a localized 15-item scale. The scale contains four dimensions: performance expectancy, effort expectancy, social influence, and facilitating conditions, measured on a 5-point Likert scale. Example items include “Digital technology improves my work efficiency” and “I find digital tools easy to use.” (*α* = 0.847).

#### 3.2.2 Job demands

The questions assessing job demands were adopted from the scale originally created by Demerouti et al. (2001). This scale comprises five questions, including the following: “My work requires a significant amount of my energy.” “I lack the requisite time to complete the task at hand” (α = 0.844).

#### 3.2.3 Digital anxiety

The study employed the measurement instrument from Firk et al. (2023). The scale comprises five items, including the following: “I worry that my personal abilities are becoming less important due to the development of digital technology” (α = 0.810).

#### 3.2.4 Workplace well-being

The study utilized the scale developed by Zheng et al. (2015), comprising six questionnaire items such as “Work is a meaningful experience for me,” “I can always find ways to enrich my work,” and “I find real enjoyment in my work” (α = 0.810).

#### 3.2.5 Control variables

The subjective emotion of workplace well-being is influenced by demographic characteristics. Building upon previous studies (Ashikali et al., 2020), this paper considers demographic characteristics, including gender, age, education, and department, as potential control variables. These variables are fully incorporated into the data analysis, enhancing the scientific rigor and credibility of the research findings.

## 4 Results

### 4.1 Statistical analysis

The research data were subjected to statistical analysis with the assistance of the statistical software packages SPSS 26.0 and Amos 24.0. First, the study evaluated the potential for common method bias, followed by an assessment of how distinct the four variables were within the hypothesized model. Second, a basic descriptive statistical analysis was conducted. Finally, the hypotheses were tested according to the procedure for mediation effect testing of the two-mediation model. The mediation effects of the main effect, job requirements, and number anxiety were examined.

### 4.2 Common method bias test

The present study employed a multitemporal data gathering strategy with the objective of reducing the likelihood of common method bias. First, Harmand’s one-way test (Podsakoff et al., 2003) was employed to factor analyze all the observed indicators of the four variables of workplace digitization, job demands, digital anxiety, and workplace well-being. The results demonstrated that multiple factors were extracted, and the first factor explained only 29.133% of the variance, which fell below the empirical standard. Furthermore, confirmatory factor analysis incorporating a common method factor was employed, that is, adding a method latent factor to the confirmatory factor model (four-factor model) (Gu and Wen, 2017). The results showed that the fit indices were not significantly improved, with changes in RMSEA and SRMR not exceeding 0.05, and changes in CFI, TLI, and IFI not exceeding 0.01. Consequently, since the sample data shows no significant common method bias, we may proceed to the subsequent statistical test.

### 4.3 Confirmatory factor analysis

To evaluate the discriminant validity of the four latent variables—workplace digitization, job requirements, digital anxiety, and workplace well-being—nested structural models were constructed to assess the model fit. Table 1 illustrates the outcomes. In the validation factor analysis of the workplace digitalization scale, the scale was divided into more topics to reduce the higher parameter estimation bias (Wu and Wen, 2011; Bentler and Chou, 1987). This study adopted the Item Parceling method (Item Parceling), which packages the multidimensional variables according to the scale dimensions. The workplace digitalization scale was divided into four packages. Among the nested models, the four-factor model outperformed the others in terms of fit indices (*χ*^2^ (382) = 396.301, χ^2^/df = 2.508, IFI = 0.928, TLI = 0.912, CFI = 0.927, RMSEA = 0.063), meeting empirical criteria for model adequacy. The indices were within an acceptable range, thereby validating the model’s hypothesis. Supported the rationality of the hypothesized model. Therefore, the four main variables in this study have high discriminant validity.

**Table 1:** Confirmatory factor analysis results.

Model	*χ* ^2^	df	χ^2^/df	IFI	CFI	TLI	RMSEA
1. Four-factor model (WD, WWB, JD, DA)	396.301	158	2.508	0.928	0.927	0.912	0.063
2. Three-factor model (WD + JD, WWB, DA)	863.484	167	5.171	0.788	0.787	0.757	0.105
3. Three-factor model (WD + DA, WWB, JD)	751.854	167	4.502	0.822	0.821	0.796	0.096
4. Three-factor model (WD, WWB, JD + DA)	686.631	167	4.112	0.842	0.841	0.819	0.09
5. Two-factor model (WD + WWB, JD + DA)	836.216	169	4.948	0.797	0.796	0.77	0.102
6. Single-factor model (WD + WWB + JD + DA)	1173.737	170	6.904	0.694	0.693	0.656	0.124

+, Two factors were combined; WD, Workplace Digitization; JD, Job Demands; DA, Digital Anxiety; WWB, Workplace well-being.

### 4.4 Descriptive statistics and correlation analysis

Here are the descriptive statistical results as presented in Table 2. Workplace digitization showed a notable negative correlation with both job demands (*r* = −0.432, *p* < 0.01) and digital anxiety (*r* = −0.516, *p* < 0.01). Job demands were also significantly negatively correlated with workplace well-being (*r* = −0.483, *p* < 0.01). Furthermore, digital anxiety was significantly and negatively associated with workplace well-being (*r* = −0.522, *p* < 0.01). The correlation between workplace digitization and workplace well-being was found to be significantly positive (*r* = 0.581, *p* < 0.01). Descriptive statistics offered initial validation for subsequent hypothesis testing. Variance Inflation Factors (VIF) for variables ranged from 1.006 to 1.427, indicating no significant problems with multicollinearity among variables.

**Table 2:** Results of descriptive statistical analysis (*N* = 382).

	*M*	SD	1	2	3	4	5	6	7	8
1. gender	1.602	0.490								
2. age	2.039	0.849	0.044							
3. edu	3.024	0.670	0.109*	−0.057						
4. age	3.628	2.089	−0.022	−0.017	−0.112*					
5. WD	4.166	0.410	−0.044	0.026	0.276**	−0.160**	(**0.847**)			
6. JD	2.402	0.806	−0.016	−0.082	−0.212**	0.148**	−0.432**	(**0.844**)		
7. DA	2.105	0.694	0.066	−0.068	−0.205**	0.221**	−0.516**	0.627**	(**0.810**)	
8. WWB	4.090	0.551	−0.031	0.109*	0.176**	−0.09	0.581**	−0.483**	−0.522**	(**0.810**)

**p < 0.01, *p < 0.05, WD, Workplace Digitization; JD, Job Demands; DA, Digital Anxiety; WWB, Workplace well-being. M=mean; SD=standard deviation. The bold values represent Cronbach’s α coefficient.

### 4.5 Hypothesis testing

Tests of the main effect and mediating effect. The present study employed stepwise regression analysis to investigate the direct and mediating effects of job demands and digital anxiety. Here are the results as presented in Table 3. Model 1 presents a model containing only control variables, while Models 2 through 6 add independent variables and mediating variables in turn. The findings from the study indicated that workplace digitization in Model 2 significantly boosted employees’ workplace well-being (*r* = 0.572, *p* < 0.001), thereby supporting the hypothesis H1. Conversely, workplace digitization in Model 8 demonstrated a marked negative impact on job demands (r = −0.393, *p* < 0.001), thereby supporting hypothesis H2a. Furthermore, the results demonstrated that job demands in Model 3 were significantly and negatively correlated with workplace well-being (*r* = −0.457, *p* < 0.001), thereby supporting the hypothesis H2b. In Model 4, the independent variable of workplace digitization and the mediating variable of job demands were introduced simultaneously. Job demands negatively correlated with workplace well-being (*r* = −0.282, *p* < 0.001). Additionally, the positive effect of workplace digitization on workplace well-being remained significant, although it exhibited a decline compared to Model 2, with a coefficient reduction from 0.572 to 0.461. This indicates that job demands partially mediate the positive effect of workplace digitization on workplace well-being. Based on the aforementioned evidence, hypothesis H2 is supported. Model 10 found that workplace digitization significantly negatively predicted digital anxiety (*r* = −0.471, *p* < 0.001), thereby supporting Hypothesis H3a. In Model 5, digital anxiety was found to significantly negatively affect workplace well-being (*r* = −0.507, *p* < 0.001), thereby supporting Hypothesis H3b. Hypothesis H3b was therefore supported. In model 6, the independent variable workplace digitization and the mediating variable digital anxiety were introduced into the equation simultaneously. The results demonstrated that digital anxiety significantly negatively affected workplace well-being (*r* = −0.306, *p* < 0.001). The findings confirmed that workplace digitization positively influenced workplace well-being, although the coefficient declined from 0.572 to 0.428 compared with model 2. This suggests that digital anxiety partially mediated the beneficial impact of workplace digitization on workplace well-being. Consequently, hypothesis H3 is corroborated.

**Table 3:** Results of multiple linear regression analysis (*N* = 382).

	Variant	WWB						JD		DA	
		Model 1	Model 2	Model 3	Model 4	Model 5	Model 6	Model 7	Model 8	Model 9	Model 10
Control variable	gender	−0.057	−0.013	−0.052	−0.018	−0.009	0.005	0.013	−0.018	0.095	0.058
	age	0.121*	0.097*	0.079	0.075	0.081	0.078	−0.092	−0.076	−0.08	−0.06
	edu	0.181***	0.025	0.087	−0.002	0.081	0.004	−0.205***	−0.098	−0.198***	−0.07
	sector	−0.069	0.006	−0.012	0.027	0.032	0.048	0.124*	0.073	0.199***	0.138***
Independent variable	WD		0.572***		0.461***		0.428***		−0.393***		−0.471***
Intermediary variable	JD			−0.457***	−0.282***						
	DA					−0.507***	−0.306***				
	R^2^	0.053	0.347	0.248	0.411	0.285	0.413	0.069	0.208	0.096	0.296
	ΔR^2^	0.053	0.295	0.195	0.063	0.232	0.066	0.069	0.139	0.096	0.199
	F	5.274***	40.048***	24.768***	43.554***	29.97***	44.055***	7.012***	19.734***	10.034***	31.564***

*N* = 382; *p < 0.05, **p < 0.01, ***p < 0.001, WD, Workplace Digitization; JD, Job Demands; DA, Digital Anxiety; WWB, Workplace well-being.

To assess the resilience of the mediating effects of job requirements and digital anxiety, the Bootstrap method was employed with the assistance of the Process plug-in in SPSS software, utilising the Mode1 4 expansion operation. The resulting Bootstrap data are presented in Table 4. The results of repeated sampling 5,000 times demonstrated that workplace digitization indirectly influenced workplace well-being through job requirements (*β* = 0.096, *p* < 0.001). The 95% confidence interval for this parameter was [0.038, 0.164], and since both the upper and lower bounds do not include 0, the findings indicate that job demands demonstrate a significant mediating effect. The present findings further substantiate the partial mediating role of job demands in the positive correlation between workplace digitization and workplace well-being, thus corroborating the proposed hypothesis H2. Similarly, workplace digitization indirectly influenced workplace well-being through digital anxiety (*β* = 0.131, *p* < 0.001). The confidence interval of this parameter at the 95% level is [0.048, 0.237], and the upper and lower intervals do not contain 0, indicating that the mediating role of digital anxiety has reached a significant level. This further supports the hypothesis that digital anxiety partially mediates the correlation between the digitization of the workplace and workplace well-being. Consequently, hypothesis H3 is validate.

**Table 4:** Bootstrap results for mediating effects.

Trails	Model	Efficiency value	Standard error	95% confidence interval	
				Lower limit	Higher limit
Workplace digitization → Job demands → Workplace well-being	Direct effect	0.542	0.063	0.418	0.667
	Indirect effect	0.096	0.322	0.038	0.164
Workplace digitization → Digital anxiety → Workplace well-being	Direct effect	0.542	0.063	0.418	0.667
	Indirect effect	0.131	0.048	0.048	0.237

## 5 Discussion

### 5.1 Conclusion

This study applied the JD-R model to investigate whether workplace digitization affects employees’ workplace well-being through job demands and digital anxiety. The findings, derived from theoretical analysis and empirical testing, indicate that workplace digitization enhances employees’ workplace well-being. Job demands negatively impact workplace well-being, and workplace digitization can enhance workplace well-being by reducing these demands. Digital anxiety negatively affects workplace well-being, and workplace digitization can enhance employees’ workplace well-being by reducing digital anxiety.

### 5.2 Theoretical contributions

Based on the JD-R model, this study expands the research scope of workplace digitization outcome variables and enriches the antecedent variables research of workplace well-being. Drawing on the JD-R model’s theoretical framework that explains how job demands deplete employee resources (loss pathway) while job resources enhance employee motivation and well-being (gain pathway) (Bakker et al., 2023), this study theoretically positions workplace digitization as a critical organizational factor that simultaneously influences both pathways. Previous research on workplace digitization has predominantly concentrated on performance-oriented outcomes such as job performance, organizational efficiency, and productivity gains (Chatterjee et al., 2023; Li et al., 2023), while overlooking employee well-being as a critical outcome variable. This study expands workplace digitization research by establishing workplace well-being as an important outcome variable at the micro-level. Simultaneously, this research enriches workplace well-being antecedent research by introducing organizational-level technological transformation as a fundamental predictor of employee well-being. In contrast to conventional studies that have predominantly concentrated on individual-level factors (personality traits, personal resources) and interpersonal factors (leadership styles, team dynamics) as antecedents of workplace well-being (Fang et al., 2023), this study establishes digitization as a macro-level organizational intervention that systematically affects employee well-being through the JD-R model’s dual pathways.

This study enriches JD-R model research by establishing a dual mediation model of job demands and digital anxiety that elucidates the complex pathways through which workplace digitization influences employee well-being. The JD-R model has found broad application in conventional work environments. For example, personality traits influence the perception of job demands and job resources, thereby affecting employee well-being (Borst and Knies, 2023). This study extends its theoretical scope and explanatory power to digitized work environments by demonstrating how technological changes simultaneously influence both job demands and job resources. Under the job demands pathway, workplace digitization reduces job demands by simplifying work processes and reducing repetitive tasks, thereby weakening the loss pathway and subsequently enhancing employee workplace well-being. Under the job resources pathway, the increased resources brought by workplace digitization promote employees’ knowledge and skill learning, strengthening the gain pathway and reducing their digital anxiety, which in turn enhances employee workplace well-being. Therefore, the dual mediation model of this study enriches the cognition and understanding of JD-R theory.

### 5.3 Management insights

Workplace digitization is an integral and crucial part of an enterprise’s digital transformation strategy. In particular, the Coronavirus outbreak has greatly accelerated this process, with digital workplaces reshaping employees’ work behaviors and work styles (de Lucas Ancillo et al., 2023). Employees’ perception and acceptance of digital transformation depend on the performance, ease of use, usability and convenience of digital tools (Ouyang et al., 2023). Therefore, organizations should actively focus on the impact of these factors during the digital transformation process. For instance, organizations can facilitate employees’ mastery of the competencies and understanding prerequisite for success in the digital work environment by providing targeted training on digital tools and technologies, thereby enhancing their productivity and well-being. In addition, regular assessment of employees’ acceptance and experience of using digital tools can be adopted in order to adjust and optimize the digital work environment in a timely manner and to promote active participation and support from employees.

In the digital transformation era, reducing employee job demands and digital anxiety is critical. In the workplace, excessive job demands can cause anxiety, job insecurity, etc. (Bakker et al., 2014; Schaufeli and Taris, 2014), and the findings highlight the adverse effects of job demands and digital anxiety on employees’ well-being in the workplace. Therefore, organizations should pay attention to employees’ workloads, allocate tasks rationally, and avoid excessive stress; concurrently, it is imperative that they prioritize the mental well-being of their employees, offering psychological counseling and other forms of support. Organizations should create a supportive work environment, encourage cooperation and communication among employees, and provide resources and support to help employees overcome the challenges that may be brought by the digital work environment and reduce their digital anxiety, so as to enhance their job satisfaction and workplace well-being.

## 6 Limitations

Firstly, the measurement of the Workplace Digitization Scale is derived from a scale developed by foreign researchers. Although the scale demonstrates satisfactory reliability and validity, further investigation is required to ascertain its conceptual connotations and to validate it in different cultural contexts. Future research could benefit from developing a specific scale to measure workplace digitization tailored to the Chinese context. For example, future research could begin with an investigation of the Chinese cultural context, employing rootedness theory to develop measurement tools for workplace digitization, job demands, digital anxiety, and workplace well-being that are more aligned with the specific characteristics of local organizations. Secondly, this study concentrated on the mechanism of influence between workplace digitization and job well-being, without examining the constraints of this mechanism. Future research could explore how specific factors like utilitarian leadership and employee self-efficacy moderate the digitization and well-being relationship. Thirdly, this study has concentrated on the positive impact pathways of digitization, and future research could further explore the potential negative effects that digitization may bring about, such as issues like increased work intensity and pressure to monitor remotely, and how organizations can balance these impacts through effective management. Finally, with regard to the collection of data, to mitigate common methodological biases, we conducted multi-temporal data collection, obtaining data on different research variables through three surveys. Although this approach partially addresses the issue of temporal causality in the theoretical model, it is still challenging to entirely eliminate the possibility of heteroscedastic error. To address this limitation, implementing varied data gathering techniques could improve the scientific and reliability of empirical testing.

## Data Availability

The raw data supporting the conclusions of this article will be made available by the authors, without undue reservation.
